# Midline non-ictal rhythmic waveforms as possible electroencephalographic biomarkers of Smith-Klingsmore syndrome in children

**DOI:** 10.1016/j.cnp.2024.02.001

**Published:** 2024-02-23

**Authors:** Valerio Simonelli, Anna Rita Ferrari, Roberta Battini, Paola Brovedani, Emanuele Bartolini

**Affiliations:** a IRCCS Stella Maris Foundation, Department of Developmental Neuroscience, Pisa, Italy; b Department of Clinical and Experimental Medicine, University of Pisa, Pisa, Italy; c Tuscany PhD Programme in Neurosciences, University of Florence, Italy

**Keywords:** EEG, MTOR, Diagnosis, Paediatric, Neurodevelopment, Genetics

## Abstract

**Introduction:**

Pathogenic variants of the MTOR gene result in the Smith-Kingsmore syndrome, whose phenotypical spectrum includes facial dysmorphisms and neurological features. Expressivity is variable, patients exhibit a combination of intellectual disability, macrocephaly and epilepsy. The diagnosis can be missed, failing to detect the causative pathogenic mutation in patients with somatic mosaicism or even skipping to analyze *MTOR* when the phenotype is not completely expressed.

**Case study:**

Herein, we report two children harboring the same MTOR recurring mutation (c.5395G>A/p.Glu1799Lys) whose EEG displayed a peculiar combination of midline rhythmic waveforms and asynchronous spike-and-wave discharges with anterior fast activity in sleep and wake. **Conclusion**: We suggest these features might be considered as possible hallmarks of the syndrome and could aid to expedite the diagnosis when the phenotype is incomplete.

## Highlights

• The SKS results from heterozygous *MTOR* mutations yielding dysmorphic traits/macrocephaly, seizures, intellectual disability.
• Phenotype is variably expressed hampering an expedite diagnosis.
• Specific electroencephalographic traits can represent endophenotypes useful to suspect SKS.

## 1 Introduction

The mechanistic target of rapamycin (mTOR) is a serine/threonine-specific protein kinase that forms two multiprotein complexes, i.e. mTORC1 – regulator of cell growth/metabolism, involved in cerebral synaptic transmission and plasticity, inhibited by rapamycin (i.e. sirolimus) – and mTORC2 – modulator of cell proliferation/survival, cytoskeletal integrity, insensitive to rapamycin. Upstream activators and repressors modulate both complexes. Germline and somatic gene variants in the mTOR cascade that provoke mTORC1 hyperactivation may cause epilepsies, malformations of cortical development and neurodevelopmental disorders (Moloney et al., 2021).

The Smith-Kingsmore syndrome (SKS) results from heterozygous gain-of-function variants of *MTOR* itself (Smith et al., 2013). The phenotypical spectrum includes (i) Noonan-like facial dysmorphisms (ii) a combination of three neurological features variably expressed: intellectual disability with impaired/absent language (92.3 %), macrocephaly/megalencephaly/hemimegalencephaly (88.9 %), seizures (73.9 %) (iii) systemic traits such as small thorax, ventriculomegaly, capillary skin malformations, hypomelanosis (Gordo et al., 2018). Diagnosis is suspected on clinical grounds and must be molecularly confirmed. Causative mutations are mostly germinal and de novo (92.6 % in Gordo et al.), yet somatic or brain-confined mosaicism has been reported (Gordo et al., 2018, Møller et al., 2016, Szczałuba et al., 2021). Inherited cases from apparently unaffected parents have been described, possibly due to gonadal mosaicism. No clear genotype-phenotype correlation is known, even though asymmetric features (hemimegaloencephaly, lateralized overgrowth) suggest mosaicism (Carli et al., 2021, Gordo et al., 2018). Mild cases that escape the typical SKS spectrum have been described, such as a mother-daughter duo with focal seizures, normal neuroimaging and cognition, no dysmorphic traits, sharing a germline variant (c.7501A.G/p.Ile2501Val) (Møller et al., 2016).

Albeit electroencephalogram (EEG) abnormalities have been reported in SKS, no typical neurophysiological features are acknowledged. Most studies have reported an admixture of non-specific and epileptiform discharges, with heterogenous and inconsistent terminology and no clear temporal or spatial pattern (“occipitoparietal spikes and slow activity, polyspike and slow waves activity, diffuse and centro-temporal epileptiform discharges, irregular slowing of background rhythms, frequent bursts of generalized slow wave convulsions”) (Gordo et al., 2018). In our report, we have sought to emphasize a peculiar EEG pattern that appeared stereotypically developed by two different patients harboring the same *MTOR* mutation, with the aim to provide a possible SKS biomarker.

## 2 Case study

**Fig. 1:**
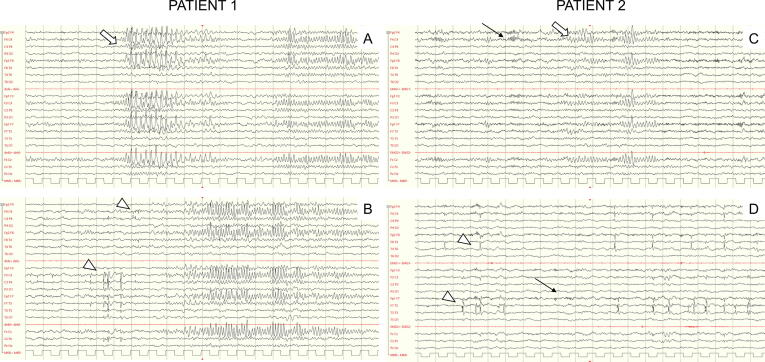
**Electroencephalographic hallmarks**. Examples of midline rhythmic waveforms (white arrows in A,C), asynchronous spike-and-wave discharges (arrowhead in B,D), anterior fast activity (thin black arrows in C,D) in drowsiness (10–20 International System, Bipolar Montage, 20-sec epoch pages. Sensitivity: 200 microV/cm; high- frequency filter: 70 Hz; low-frequency filter 1 Hz).

Patient 1 is a 10-year-old boy carrying a de novo *MTOR* pathogenic missense variant (c.5395G>A/p.Glu1799Lys) with a history of developmental delay. He started to pronounce intelligible words at 18 months of age and to walk autonomously 6 months later. We first evaluated him when aged 7 years and obtained the molecular diagnosis one year later. He exhibited macrocrania (+3 SD), muscular hypotonia, motor clumsiness, attention liability, sialorrhea, strabismus and minor dysmorphic traits (dolichocephaly, facial hypotonia). No systemic features were present. The neuropsychological evaluation disclosed mild intellectual disability (QIT = 57) as well as a Communication Disorder affecting language and speech-sound production (Supplementary Material). On brain MRI we found megalencephaly, corpus callosum dysmorphism (absent rostrum, hypoplasia of the splenium) and right perisylvian polymicrogyria. The electroencephalogram (EEG) depicted a peculiar pattern in the wake, enhanced in sleep, composed by rhythmic notched sharp wave with a wax-and-wane appearance on the anterior midline electrodes; asynchronous centrotemporal and temporo-occipital epileptiform discharges and anterior fast activity co-occurred (Fig. 1). No seizures have risen during follow-up, and the electroencephalographic features have persisted unchanged to date.

Patient 2 is an 8-year-old child, born by medically assisted reproduction (oodonation), harboring the same *MTOR* pathogenic variant as Patient 1 – absent in the father, presumed to be de novo (c.5395G>A/p.Glu1799Lys). Development was mildly delayed; disyllabic words started to be pronounced at 12–18 months, autonomously ambulatory at 18 months of age. Aged 4-year-old, febrile seizures occurred. Attention and language difficulties set in. A brain MRI revealed dysmorphism of the corpus callosum, which appeared shortened and thickened at the genu level. A molecular diagnosis was obtained at age 6-year-old. Two years later he was referred to our Institute. We diagnosed mild intellectual disability (QIT = 61), a Communication Disorder with language and speech/sound impairment, attention deficit and hyperactive disorder (Supplementary Material). We found macrocephaly (+3 SD) but no facial dysmorphism or other systemic features. The EEG depicted similar features to Patient 1, namely rhythmic sharply contoured waves on the midline; asynchronous spike and sharp waves on the right frontocentral and left temporal regions as well as anterior fast activity in sleep and wake were also present. After febrile seizures in infancy, no other paroxysmal events have been noted until now.

## 3 Discussion

Mutations of genes involved in the mTOR pathway are especially associated with epilepsy, macrocephaly, intellectual disability. Each ‘mTORopathy’ may exhibit distinctive features. Noonan-like facial dysmorphisms (e.g. curly hair, frontal bossing, tall forehead, hypertelorism, down slanting palpebral fissures, bitemporal narrowing, macrostomia, short nose with flat nasal bridge and long philtrum) can represent diagnostic hints for SKS when identified in patients with seizures, macrocephaly and intellectual disability. Nevertheless, patients may display an incomplete phenotype or even features overlapping with other ‘mTORopathies’ such as capillary malformations similar to those observed in Megalencephaly-capillary-Malformation (mutations of the upstream regulator: *PIK3CA* gene) (Mirzaa et al., 2016).

Our patients shared the very same pathogenic variants and exhibited two cardinal clinical signs: macrocephaly and intellectual disability with language impairment. Only Patient 1 displayed facial dysmorphic traits, that however were very mild. Neither of them had epilepsy, even though Patient 2 had experienced febrile seizures in early childhood.

In SKS, the diagnosis can be missed, failing to detect the causative pathogenic mutation in patients with somatic mosaicism, or even skipping to analyze *MTOR* when the phenotype is not completely expressed. Indeed, the genotype-phenotype correlation is poor: patients with germinal mutations may exhibit mild signs, and those with somatic pathogenic variants can develop a full-blown phenotype. Neuroimaging can also be not specific. Patients with germinal mutations may have normal imaging or minor abnormalities such as dysmorphism of the corpus callosum – as we observed – but also overt megalencephaly; somatic mutations most often promote more sectorial malformations such as focal cortical dysplasia (Mirzaa et al., 2016, Møller et al., 2016).

Of note, we caught peculiar electroencephalographic traits that may represent possible endophenotypes useful to suspect SKS. Former works have not specifically addressed the EEG pattern (Gordo et al., 2018). So far, no particular EEG abnormality has been highlighted. In the first description of SKS, Smith et al. described a newborn with relentless non-motor apneic seizures, megalencephaly and dysmorphic features, who deceased at 19 months of age due to respiratory complications, harboring a missense pathogenic variant (c.4448G>T p.Cys1483Phe). The EEG assessment suggested an encephalopathic trajectory, with recurrent focal electrographic seizures at onset (2-month-old: high voltage spike and slow wave activity in the left occipital-temporo-parietal region) and abundant bilateral interictal multifocal discharges during follow-up (8-month-old) (Smith et al., 2013). Strikingly, patients with low-level brain mosaicism may also develop encephalopathic features. Szczałuba et al. reported hypsarrhythmia in an infant with neonatal drug-resistant seizures, recurrent episodes of hypoglycemia, and facial dysmorphisms, who underwent surgical resection for hemimegaloencephaly. The genetic analysis of the surgical specimen revealed a pathogenic variant restricted to the brain (c.6644C>T p.(Ser2215Phe). Post-operatively, seizures persisted unchanged and an overt neurodevelopmental impairment set in (Szczałuba et al., 2021). These cases may represent the most severe end of the phenotypical spectrum; repeated focal seizures in a rapidly worsening EEG might suggest a dismal prognosis. Such an unfavorable EEG and clinical evolution is likely rare and driven by the underlying molecular defect rather than the presence of a brain malformation. In fact, Carli et al. described a single case with somatic mosaicism (c.4448G> A, p.Cys1483Tyr) who displayed lateralized overgrowth with ipsilateral hemimegalencephaly, whose seizures were drug-responsive and the EEG showed “sporadic anomalies” (Carli et al., 2021). Gordo et al. reviewed published cases of SKS, either with germinal or somatic mutations, generically reporting ‘irregular slowing of background rhythms’ in isolated reports (Gordo et al., 2018). In patients with the same variant of ours, Poole et al. generically described afebrile seizures in 5/16 patients (31 %), recurring in only 2 individuals to suggest a favorable course; no specific EEG features were described, except generalized epileptiform discharges in one subject (Poole et al., 2021).

In our experience, midline rhythmic waveforms in wake and sleep arose as cardinal characteristics. These had a sinusoidal and sharply contoured appearance and partially resembled ‘Brief Potentially Ictal Rhythmic Discharges (BIRDs)’ and ‘6-Hz spike and slow wave (phantom spike-waves)’. BIRDs were initially described in newborns as very brief (<10 s) runs of rhythmic activity of >4 Hz, with or without evolution. However, the BIRD pattern hasn’t a midline predilection but is typically lateralized or generalized. BIRDs have been described in neonates with a not specific encephalopathy, in the critically-ill adult or in people with drug-resistant epilepsy (Yoo et al., 2017), whilst our Patients had none of these features. Phantom spike-waves are bursts of fast spike-and-waves (5–7 Hz for 1–2  s) with low-voltage and short spikes (<25 µV, <30  ms), typically triggered by drowsiness, rare in children, more frequently found in adulthood; some Authors interpret Phantom spike-waves as benign variants, yet they might be associated to epilepsy when predominating over the anterior regions (Hughes, 1980). Conversely, the midline non-ictal rhythmic waveforms we found did not vary with alertness and appeared very similar between two Patients of pediatric age with the same pathogenic mutation, reasonably representing a proper phenotypical trait.

We also found two additional EEG striking features: asynchronous spike-and-wave discharges and anterior fast activity in sleep and wake. Multifocal epileptiform discharges confirm seizure propensity. Epilepsy is not inevitable, considering about 15 % of patients with SKS do not exhibit seizures, yet a careful follow-up with repeated EEG is mandatory. No studies have systematically addressed the seizure semiology. Convulsions or infantile spasms have been anecdotally described and are usually easily recognizable by caregivers. Focal impaired-awareness seizures might be more insidious and not readily identifiable in patients who are also often affected by intellectual disability.

The *MTOR* mutation of our Patients (c.5395G>A/p.Glu1799Lys) is indeed recurrent and can be considered a mutational hotspot, occurring in about half of SKS patients (Gordo et al., 2018).

Functional studies have demonstrated that most pathogenic *MTOR* variants – including c.5395G>A/p.Glu1799Lys - disrupts the alpha-helix packing in the inhibitory FAT domain of the protein, resulting in gain-of-function with enhancement of both mTORC1 and mTORC2 activity with disruption in the formation of neural circuitry (Besterman et al., 2021, Grabiner et al., 2014, Mirzaa et al., 2016, Møller et al., 2016). Colon polyposis and kidney cysts have also been described (Moosa et al., 2017). The glutamic acid substitution in the *MTOR* gene at position 1799 – as in our cases- has been encountered as a recurrent somatic mutation site in several cancer tissues, even though the long-term risk of cancer development is currently unknown (Grabiner et al., 2014). We should consider that the activation of the mTOR pathway can be involved in cancerogenesis, of which vivid example is the PTEN-hamartoma-tumor-syndrome wherein the screening for colorectal, renal, thyroid, breast, endometrial and skin is recommended since 18 years of age (Tischkowitz et al., 2020). Tuberous sclerosis complex, in which *TSC1*/*TSC2* mutations activate the mTOR pathway, is similarly burdened by oncological risks (e.g. subependymal giant astrocytomas, renal angiomyolipomas, cardiac rhabdomyomas) (Northrup et al., 2021).

A prompt identification of SKS electroencephalographic biomarkers could expedite the diagnosis paving the way to precision medicine. In the Tuberous Sclerosis Complex, everolimus (mTOR inhibitor) is effective in reducing the size of subependymal giant astrocytomas and renal angiomyolipomas and in improving the seizure frequency (Northrup et al., 2021). The benefit of mTOR inhibition may also be applicable to other ‘mTORopathies’ such as SKS, yet further studies are needed to corroborate this hypothesis.

Our findings require to be confirmed in larger cohorts and compared to subjects harboring different mutations, who have been anecdotally reported to display more severe EEG features. The rarity of the disorder calls for a multicenter collaboration to obtain generalizable results. Prospective studies would be especially needed to assess whether EEG abnormalities can be age-dependent, follow a dynamic time course, and bring a higher risk of seizures in the long-term.

## Funding

This work has been partially supported by grant-RC and the 5x1000 voluntary contributions, 10.13039/501100003196Italian Ministry of Health.

## Ethical approval

Written parental informed consent was obtained for the description of the index cases. All the procedures complied with the Helsinki Declaration.

## Declaration of competing interest

The authors declare that they have no known competing financial interests or personal relationships that could have appeared to influence the work reported in this paper.
