# Supplementary material for: Midline non-ictal rhythmic waveforms as possible electroencephalographic biomarkers of Smith-Klingsmore syndrome in children
Source: Clin Neurophysiol Pract. 2024 Feb 23;9:102–5. doi: 10.1016/j.cnp.2024.02.001 (PMC10940733; doi:10.1016/j.cnp.2024.02.001)
Supplement: Supplementary Data 1 [file mmc1.docx]

**SUPPORTING MATERIAL**

**Neuropsychological phenotyping**

PATIENT 1: Patient 1 is a 10 year-old boy carrying a de novo MTOR pathogenic missense variant (c.5395G>A/p.Glu1799Lys). He presented feeding difficulties and drooling and had delayed language milestones. He started to pronounce the first words at 18 months of age with successive difficulties in lexicon and morphosyntax organization. He walked autonomously 6 months later. During childhood, cognitive difficulties, motor clumsiness and attention lability set in. We first evaluated the patient at age 7 years. The boy presented with mild Intellectual Developmental Disorder (DSM V-TR, 2022) with a Full scale IQ of 57 (WPPSI-III, Wechsler, 2002) and an Adaptive Behaviour Composite of 62 (Vineland II, Sparrow et al., 2005). A mild Communication Disorder (DSM V-TR, 2022) was present at the time affecting language (expressive and receptive skills) as well as speech-sound production (reduced phonetic inventories and articulation difficulties) and social skills (turn taking, echolalia). Verbal IQ (62) and Performance IQ (65) did not differ at the time, but there was a significant drop in verbal intelligence (Verbal Comprehension Index 46) with respect to non-verbal skills ( Perceptual Reasoning Index 52) at follow up with the WISC-IV (Wechsler, 2003) at age 8 years. At follow-up, spontaneous language was present, but he showed a persistence of speech-sound production difficulties albeit with a reduction of drooling, and simplified grammar.

PATIENT 2: Patient 2 is an 8 year-old child, born by medical assisted reproduction with oodonation, harbouring the same MTOR pathogenic variant as Patient 1 (c.5395G>A/p.Glu1799Lys) – absent in the father-, presumed to be de novo. At birth, macrocephaly, camptodactily and hydrocele were noted. Development was mildly delayed, with emergence of two-syllable words at 12-18 months and autonomous walking at 18 months. At 4 years of age, febrile seizures occurred, and shortly after, attention and language difficulties arose. Intelligence testing revealed mild intellectual impairment with a Full-Scale IQ of 66 (WPPSI-III). Brain MRI revealed dysmorphisms of the corpus callosum which appeared shortened and thickened at the genu level. Exome sequencing revealed the MTOR mutation at age 6 years. Two years later, he was referred to our Institute. He presented with a moderate Intellectual Developmental Disorder with a Full-Scale IQ of 61 (WPPSI-III) and an Adaptive Behavior Composite of 62 (Vineland II) associated with a Communication Disorder affecting Language (expressive skills, especially grammatical control and receptive skills), and speech sound production (speech not fully intelligible, stuttering). There was no difference between verbal and non-verbal intelligence (Verbal IQ 60; Performance IQ 59). A comorbidity with ADHD (Combined presentation) was also formulated. Motor stereotypies, restricted interests, insistence on sameness and inflexible adherence to routines were reported by parents. The patient did not fulfil criteria for Autism Spectrum Disorder).

The boy presented with macrocephaly (+3 SD) without facial dysmorphisms nor other systemic features. The EEG depicted similar features to Patient 1, namely rhythmic sharply contoured waves on the midline augmented by hyperventilation and drowsiness, anterior fast activity in wake/sleep and asynchronous spike and sharp waves on the right frontocentral and left temporal regions in sleep. After febrile seizures in infancy, no other paroxysmal events have been noted to date.

**Supporting Table S1**

Summary of clinical features, epilepsy characteristics and EEG features of previously published SKS original reports

| **Authors** | **Patients** | **Clinical features** | **Variant** | **Seizures/**  **Epilepsy** | **Type of seizures** | **Seizure onset** | **EEG** |
| --- | --- | --- | --- | --- | --- | --- | --- |
| **Smith et al.** | F | Macrocephaly,  frontal bossing, prominent forehead, downslanting palpebral fissures,  long eyelashes, short upturned nose, umbilical hernia, and myopathic  appearance. | c.4448 G>T (p.Cys1483Phe) | 1/1 | Apnea  episodes initially not associated with motor involvement | 2 M | Focal electrographic seizures at onset: High voltage  (0.5–1.5 Hz) spike and slow wave activity with evolution to faster 2 to 3 Hz spike and slow wave discharges in the left occipto-temporo-parietal region |
|  |  |  |  |  |  |  |  |
| **Baynam et al.** | F  M  M | Macrocephaly, ID, craniofacial features, café-au-lait lesion, renal asimmetry  Macrocephaly, ID, craniofacial features, café-au-lait lesion, renal asimmetry  Macrocephaly, ID, craniofacial features, café-au-lait lesion | c.5395G>A (p.Glu1799Lys) in all probands | 3/3 | NA | NA | NA |
| **Mroske et al.** | M  M | Megalencephaly, ID, iris colobomas, strabismus, hypotonia, ASD  Megalencephaly, ID, iris colobomas, hypotonia, ASD, undescended testes | c.5395G>A (Glu1799Lys) in both probands | 0/2 | NA | NA | NA |
| **Moose et al.** | F  M | Macrocephaly, craniofacial features, ID, multiple polyps in the ileum, cecum and colon  Macrocephaly, craniofacial features, ID | c.5395G>A p.(Glu1799Lys) in both probands | 0/2 | NA | NA | NA |
| **Gordo et al.** | M  M  F  M | Megalencephaly, ASD, ID, hyperactivity  Megalencephaly, ID  Megalencephaly, ID, seizures  Megalencephaly, ID | c.5395G>A (p.Glu1799Lys)  c.4448G>A (p.Cys1483Tyr)  c.6605T>G (p.Phe2202Cys)  c.6605T>G (p.Phe2202Cys) | 1/4 | NA | NA | NA |
| **Rodriguez-Garcìa et al.** | F | Macrocephaly, dysmorphic facial features, developmental delay, hypotonia, combined oxidative phosphorylation deficiency, epilepsy and anti-phospholipid antibodies (aPL) | c.7235A>T (p.Asp2412Val) | 1/1 | NA | NA | NA |
| **Elizondo Plazas et al.** | M | Macrocephaly, cryptorchidism, developmental delay | c.5663T>G (p.Phe1888Cys) | 0/1 | NA | NA | NA |
| **Poole et al.** | 16 |  | c.5395G>A (Glu1799Lys) in all probands | 5/16 | Single nocturnal tonic clonic seizure;  focal then tonic clonic seizures; one seizure | NA | normal (n=2); generalized discharges (n=1) |
| **Carli et. al** | M | hemimegalencephaly, mild developmental delay, lateralized overgrowth, and Ito hypomelanosis | c.4448G > A, p. (Cys1483Tyr) | 1/1 | Generalized tonic seizures | 18 months | Sporadic anomalies |
| **Szczaluba et. al** | M | developmental delay, global hypotonia, hemimegalencephaly with pachygyria and polymicrogyria, streaks of hyper-/hypopigmentatio, history of hypoglycemia | c.6644C>T; p (Ser2215Phe) | 1/1 | Lower-limb clonic episodes, oculomotor attacks and oral automatisms | 1 month | Hypsarrhythmia |

**♦** NA: not available. N: not present. M=month/s. Y: year/s. ID = intellectual disability. ASD = autism spectrum disorder

**Additional references**

American Psychiatric Association (2022): Diagnostic and Statistical Manual of Mental Disorders, Fifth Edition, Text Revision. Washington, DC, American Psychiatric Association.

Baynam G, Overkov A, Davis M, et al. A germline MTOR mutation in Aboriginal Australian siblings with intellectual disability, dysmorphism, macrocephaly, and small thoraces. Am J Med Genet A. 2015; 167: 1659– 1667.

Carli D, Ferrero GB, Fusillo A, Coppo P, Selva RL, Zinali F, et al. A new case of Smith-Kingsmore syndrome with somatic MTOR pathogenic variant expands the phenotypic spectrum to lateralized overgrowth. Clinical Genetics. 2021;99(5):719–23.

Crino PB. The mTOR signalling cascade: paving new roads to cure neurological disease. Nat Rev Neurol. 2016 Jul;12(7):379–92.

Elizondo-Plazas A, Ibarra-Ramírez M, Garza-Báez A, Martínez-de-Villarreal LE. Expanding the phenotype of MTOR-related disorders and the Smith-Kingsmore syndrome. Neurol Genet. 2020 May 7;6(3):e432. doi: 10.1212/NXG.0000000000000432.

Forbes SA, Beare D, Gunasekaran P, Leung K, Bindal N, Boutselakis H, et al. COSMIC: exploring the world’s knowledge of somatic mutations in human cancer. Nucleic Acids Res. 2015 Jan;43(Database issue):D805-811.

Hirsch LJ, Fong MWK, Leitinger M, LaRoche SM, Beniczky S, Abend NS, et al. American Clinical Neurophysiology Society’s Standardized Critical Care EEG Terminology: 2021 Version. J Clin Neurophysiol. 2021 Jan 1;38(1):1–29

Hughes JR. Two forms of the 6/sec spike and wave complex. Electroencephalography and Clinical Neurophysiology. 1980 May 1;48(5):535–50.

Mroske C, Rasmussen K, Shinde DN, et al. Germline activating MTOR mutation arising through gonadal mosaicism in two brothers with megalencephaly and neurodevelopmental abnormalities. BMC Med Genet. 2015; 16: 102.

Rodríguez-García ME, Cotrina-Vinagre FJ, Bellusci M, Martínez de Aragón A, Hernández-Sánchez L, Carnicero-Rodríguez P, Martín-Hernández E, Martínez-Azorín F. A novel de novo MTOR gain-of-function variant in a patient with Smith-Kingsmore syndrome and Antiphospholipid syndrome. Eur J Hum Genet. 2019 Sep;27(9):1369-1378. doi: 10.1038/s41431-019-0418-1.

Sparrow, S. S., Cicchetti, D., Balla, D. A. (2005). Vineland-II: Vineland adaptive behavior scales (Second Edition). Minneapolis, USA: Pearson.

Wechsler, D. (2002). The Wechsler Preschool and Primary Scale of Intelligence, Third Edition (WPPSI-III). San Antonio, TX, USA: The Psychological Corporation

Wechsler, D. (2003). Wechsler Intelligence Scale for Children-Fourth Edition. WISC-V: San Antonio,TX, USA, The Psychological Corporation.

Wechsler, D. (2012). Wechsler preschool and primary scale of intelligence-Fourth Editions (WPPSI-IV), San Antonio, TX, USA: Pearson.
